# Evaluation of Healthcare Use Trends of High-Risk Female Intimate Partner Violence Victims

**DOI:** 10.5811/westjem.2014.12.22866

**Published:** 2015-01-05

**Authors:** Robyn M. Hoelle, Marie-Carmelle Elie, Emily Weeks, Nancy Hardt, Wei Hou, Hui Yan, Donna Carden

**Affiliations:** * University of Florida, Department of Emergency Medicine, Gainesville, Florida; † University of Florida, Family Data Center, Gainesville, Florida; ‡ Stony Brook University Medical Center, Department of Preventative Medicine, Stony Brook, New York; § University of Florida, Department of Biostatistics, Gainesville, Florida

## Abstract

**Introduction:**

Practitioners need more information about intimate partner violence (IPV) victims’ healthcare use trends. We used a novel data-linkage method and complaint categorization allowing us to evaluate IPV victims healthcare use trends compared to the date of their victimization.

**Methods:**

This was a retrospective case series using data-linking techniques cross-referencing databases of Medicaid-eligible women between the ages of 16 and 55 years, an IPV Case Database for 2007 and the Florida State Agency for Healthcare Administration, which tracks hospital inpatient, ambulatory and emergency department (ED) use within the State of Florida. We analyzed resulting healthcare visits 1.5 years before and 1.5 years after the women’s reported IPV offense. Using all available claims data a ‘complaint category’ representing categories of presenting chief complaints was assigned to each healthcare visit. Analysis included descriptive statistics, correlation coefficients between time of offense and visits, and a logistic regression analysis.

**Results:**

The 695 victims were linked with 4,344 healthcare visits in the four-year study period. The victims were young (46% in the 16–25 age group and 79% were younger than 35). Healthcare visits were in the ED (83%) rather than other healthcare sites. In the ED, IPV victims mostly had complaint categories of obstetrics and gynaecology-related visits (28.7%), infection-related visits (18.9%), and trauma-related visits (16.3%). ED use escalated approaching the victim’s date of offense (r=0.59, p<0.0001) compared to use of non-ED sites of healthcare use (r=0.07, p=0.5817). ED use deescalated significantly after date of reported offense for ED visits (r=0.50, p<0.0001) versus non-ED use (r=0.00, p=0.9958). The victims’ age group more likely to use the ED than any other age group was the 36–45 age group (OR 4.67, CI [3.26–6.68]).

**Conclusion:**

IPV victims use the ED increasingly approaching their date of offense. Presenting complaints were varied and did not reveal unique identifiers of IPV victims. This novel method of database matching between claims data and government records has been shown to be a valid way to evaluate healthcare utilization of at-risk populations.

## INTRODUCTION

Intimate partner violence (IPV) occurs in two to five million intimate partner relationships in the United States each year.^1^,^2^ Healthcare use and costs are high during and after the abuse.^3^,^4^ Several studies demonstrate that women experiencing IPV are more likely to use the emergency department (ED) and hospital resources.^5^–^9^ In one study, 18% percent of the victims in EDs reported seeking medical attention because of the abuse.^10^ Frequently, female IPV victims present with a broad range of healthcare complaints rather than abuse-related traumatic injuries.^11^–^13^ While IPV victims are likely to receive routine healthcare, they are also more likely to have been treated for an injury compared to other women.^10^ IPV victim advocates consider these healthcare encounters valuable opportunities to identify and potentially intervene on behalf of the victim.

The practice of universal IPV screening during clinical encounters, however, remains controversial due to the extensive resources required to implement successful IPV screening tools and intervention programs. Such implementation is especially challenging in busy clinical environments such as the ED.^1^,^14^ To create and sustain resourceful and cost-efficient programs, guidance is needed regarding the healthcare use practices of IPV victims including locations and types of treatments sought. Providers can assist in identifying IPV victims through recognition of use patterns or avoid under-diagnosis by relying on non-evidenced based methods. Further analysis of the timing of the index assault in the context of a clinical encounter may also provide important guidance regarding when healthcare providers have the greatest opportunity to intervene.

The methodology used to generate contemporary reports of IPV epidemiology are limited and can suffer from bias. Investigations are frequently based on a single healthcare encounter, rely on victim self-report, or legal convictions used in victim identification.^15^–^17^ Factors such as the presentation of IPV victims to multiple healthcare facilities, the reluctance of victims to report abuse and the low ratio of abuse to legal conviction rate confound the accurate characterization of IPV and the rate of victim healthcare use. Despite these shortcomings, several studies have identified that women classified as receiving public assistance or in a lower socioeconomic group have a high prevalence of IPV.^18^,^19^ The study population of Medicaid-eligible women are classified in lower socioeconomic groups in Florida and provided an accessible database for necessary cross-referencing. The healthcare use patterns of these women can be longitudinally tracked through administrative data. Further, the Florida State Attorney’s Office of Victim Witness Services maintains a database of IPV victims obtained through sworn complaints, sexual assaults, arrests and IPV homicides within a six-county area. This database of IPV victims is much broader than those requiring legal conviction.

Visit-level information related to healthcare encounters with IPV victims is rarely reported: a critical factor in enhancing the recognition of IPV patients by advocates and healthcare providers. While analysis of diagnoses and population characteristics is important for recognizing healthcare use patterns, visit-level complaint data can assist physicians in recognition of IPV victims prior to final diagnosis. This study uses a complaint category-based assessment of IPV victims’ visits to provide a more relevant evaluation of their presentation patterns to healthcare providers. Knowing presentation patterns of IPV victims can help emergency physicians with pattern recognition of victims, as well as dispel myths about IPV victims. The objective was to characterize healthcare use patterns in female IPV victims who were Medicaid-eligible and between the ages of 16 and 55 identified by the Office of Victim Witness Services in the Florida State’s Attorney’s Office (SAO), Eighth Judicial Court using database-linking methods.

## METHODS

### Study Design

We conducted a retrospective case series using data-linking techniques, cross-referencing databases of the Florida State Attorney’s Office (SAO) of Victim Witness Services 2009 IPV victim database, Medicaid-eligible women between the ages of 16 and 55 years, and the Florida State Agency for Healthcare Administration, which tracks hospital inpatient, hospital-based ambulatory, and ED use within Florida. The local institutional review committee approved this study.

### Study Setting and Population

Our cohort included Medicaid-eligible female IPV victims identified through the State of Florida Attorney’s Office of Victim Services in northern Florida including a six-county area. The women in our cohort were females in the SAO’s adult IPV incident database whose IPV offense occurred between January 1, 2007 and December 31, 2007, and were between 16 and 55 years old. Victims included in the database were identified by law enforcement officers or victim advocates. Responding officers or victim advocates evaluated daily “offenses”: sworn complaints, sexual assaults, arrests and IPV-related homicides. ”Sworn complaints” are calls police officers receive by victims or bystanders that warrant a visit. For example, if a neighbor calls that he hears yelling next door, and the police investigate and determine the situation to be IPV-related, this incident will be reported as an IPV-related sworn complaint, and added to the SAO’s IPV database. Each law enforcement interaction, within the six counties of the Eighth Circuit Court, that the responding officer or victim advocate suspects to be IPV-related, are submitted to the SAO Office of Victim Services. The SAO reviews all reports and deems the events to be IPV-related or not. Seeking out these officer- or advocate-identified incidents ensured a variety of types of IPV and severities of IPV-related victimization were included in the study rather than relying on higher-level court-determined incidents to validate IPV events. This created an inclusive population of the confirmed IPV victims recognized in this six-county region to study.

### Study Protocol

This cohort of confirmed IPV victims were linked to their healthcare visits within the state of Florida through the state’s Agency for Healthcare Administration (AHCA) database records 1.5 years before and up to 1.5 years after the IPV victim’s first IPV incident (index event). Researchers used a third-party data management group which received the list of victims from the SAO and cross-linked the victim identifiers to government databases. Their techniques involved a software program matching IPV victims to Medicaid-eligible women by first name, last name, and date of birth. The Social Security number from this cohort of women was then used to query the AHCA claims database. De-identified data was then delivered to the researchers by assigning each victim a unique identifier. These linkages resulted in robust claims data for each of the linked IPV victims.

The database includes financial, procedural and diagnostic data for all inpatient stays, ED visits, inpatient psychiatric visits, rehabilitations stays, and hospital-based ambulatory care medical records throughout the state. Each unique visit identified (most victims had several visits) was assigned a complaint category in order to evaluate trends in the women’s presenting complaints over time. To assign this complaint category, researchers reviewed the claims data for each visit including patient diagnostic codes, reason for admission codes, reason for injury codes, and procedure codes. Category assignments included trauma, infectious, obstetric, gynecologic, dental, ophthalmologic, hematologic, endocrine, cancer, psychiatric, pulmonary, cardiac, gastroenterologic, neurologic, drugs/intoxication, orthopedic, dermatologic, and ears/nose/throat. We categorized all reproductive-related complaints as obstetric- or gynecologic-related, including genital infections, instead of including these visits in the infectious category. Acute infectious complaints such as pneumonia, pharyngitis, or cellulitis were categorized to the infection-related complaints. For example, a visit with codes indicating vaginal bleeding would be categorized as a gynecology-related visit, and a visit with a diagnostic code indicating retinal tear would be categorized as an ophthalmology-related visit, while a visit indicating orbital cellulitis would be categorized under infectious. We constructed these categories to closer represent presenting complaints categories of ED patients to help identify trends in the undifferentiated patient as opposed to relying on the final diagnostic code evaluation. By tracking these complaint categories we hope to establish whether IPV victims present with complaints of one type prior to their date of offense more often than other complaints regardless of their traditional association with IPV.

### Outcome Measures

Outcome measures included healthcare use patterns. These specific variables included date of visit, site of visit, and reason for healthcare resource use, including complaint category of visit compared to date of offense.

### Data Analysis

We performed descriptive analysis for all variables, and median and IQR were reported for quantitative measures. We classified date of healthcare visit in relation to the date of reported offense, by number of weeks prior to or after the occurrence. This allowed for comparison of overall trends in healthcare use across the cohort of patients. Researchers compared dates of healthcare visits to the time of offense by calculating correlation coefficients to compare time interval and type of visits to the date of offense. We performed a logistic regression analysis comparing victims who used the ED versus victims who used other healthcare sites. Data was analyzed using SAS version 9.4 (Cary, NC), and a p-value less than 0.05 was considered significant.

## RESULTS

### Descriptive Data

There were 695 separate IPV offenses identifying 695 unique IPV victims within the six-county area, aged 16–55 years old, identified as victims of IPV by the State of Florida Attorney’s Office in 2007. The cohort of 695 Medicaid-eligible IPV victims resulted in a total of 4,344 statewide healthcare visits found in 1.5 years before and after each victim’s identifying offense. The median number of healthcare visits per victim was four (IQR=6), and the median number of ED visits per victim was three (IQR=5). However, there was great variability among victims as indicated by the IQR. The number of healthcare visits per IPV victim ranged from one visit to 98 visits. Fifty-three percent of the visits were before the date of the victim’s offense versus 47% after the date of the victim’s offense. Many victims fell into the 16–25 year old age group (46% of the victims), indicating a relatively young study population. Overall, 79% of the victims were 35 years old or younger at the time of the healthcare use. Most victims were Caucasian (52%) or African American (46%), reflecting the population of the study state.

Eighty-three percent of the total 4,344 healthcare visits by IPV victims occurred in the ED. Considering all healthcare visits, the most common complaint categories were obstetric-gynecology-related visits (28.7%), followed by infection-related visits (18.9%), and trauma-related visits (16.3%). Among only ED healthcare visits, the most common complaint categories of IPV victims were infection (22.4%), trauma (19.4%), and obstetric-gynecologic (18.8%).

### Correlations Data

**Figure 1:**
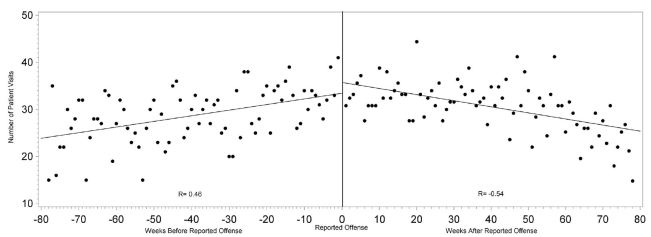
Correlation between number of all healthcare visits and the time from each intimate partner victim’s reported date of offense.

**Figure 2:**
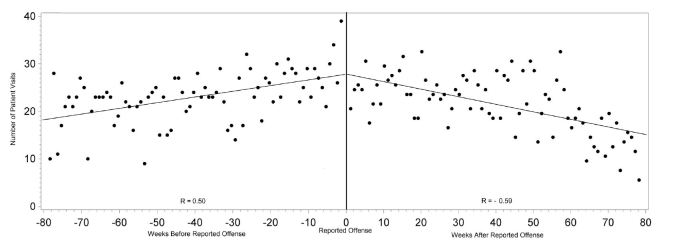
Correlation between number of emergency department visits and the time from each victim’s reported date of offense.

**Figure 3:**
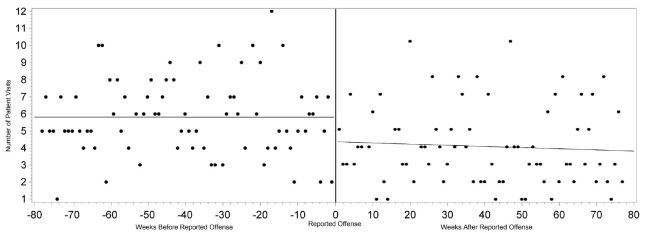
Correlation between number of non-emergency department visits and the time from each victim’s reported date of offense.

Overall healthcare use by victims escalated approaching their individual dates of reported offenses, with a moderately positive linear correlation (r=0.46, p<0.0001). ED visits also demonstrated a strongly strong positive linear correlation of escalating visits approaching the date of reported offense (r=0.50, p<0.0001) compared to non-ED healthcare visits (r=0.00, p=0.9958). Both total healthcare visits (r=−0.54, p<0.0001), and ED visits (r=−0.59, p<0.0001) demonstrated a strong linear correlation with declining visits after the date of reported offense compared to non-ED healthcare visits (r=−0.07, p=0.5817) (Figure 1–3).

**Figure 4:**
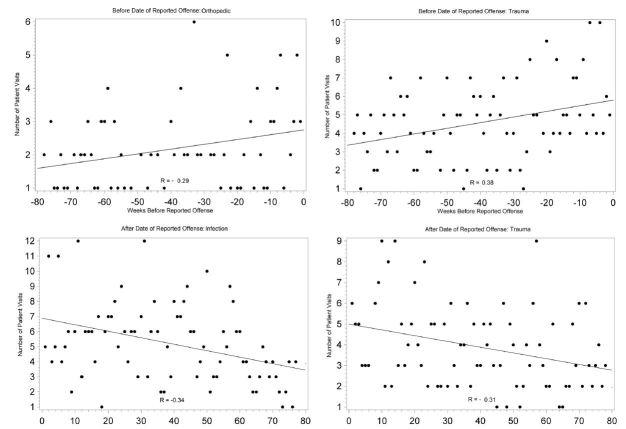
Correlation between number of all healthcare visits and the time from each victim’s reported date of offense within specific complaint categories.

Among all healthcare visits, those with the assigned complaint category of orthopedic (r=0.28, p=0.0266) and trauma (r=0.34, p=0.0024) had positive weak correlations with increasing number of visits up to the date of reported offense. Complaint categories with a weak correlation of declining visits following the day of the reported offense include trauma (r=−0.31, p=0.0060) and infection (r=0.38, p=0.0008) (Figure 4). While individuals within some of the smaller groups of complaints, like hematologic and endocrine, had significantly increasing visits up to or after date of offense, cohorts lacked power to report as an overall healthcare trend.

Psychiatric complaint category-related visits before (r=0.10, p=0.5347) and after (r=0.06, p=0.7202) date of reported offense, were not correlated with the time of reported IPV offenses. None of the other complaint categories had significant correlations to or from the time of reported offense.

### Logistic Regression Data

We compared victims who used the ED to victims who chose non-ED healthcare settings. Victims were 40% more likely to use ED healthcare settings after the date of reported offense versus before, with an OR of 1.41 (95% CI [1.20–1.66], p<0.0001). The age group more likely to use the ED versus non-ED healthcare settings was the 36–45 age group compared to the youngest group of women (OR 4.67, CI [3.26–6.68]). There were no significant differences between races in presenting in the ED versus non-ED healthcare settings.

## DISCUSSION

This study offers healthcare providers insight into the healthcare use of IPV victims through an expanded analysis of a unique inclusive cohort of IPV victims’ healthcare use. Most studies are limited to retrospective reviews of court-identified or self reports of victims and small local populations.^15^,^16^,^20^,^21^ The first way this study is unique is that our data represent statewide-claims data capturing statewide healthcare use by the confirmed IPV victims in the six-county area. Secondly, the cohort is distinct, including victims identified through sworn complaints, which is a call to the police or any police-reported IPV, and includes identification of IPV-related events prior to more severe occurrences (ie. arrest or fatality). One prior study (n=3,333), focusing on population-based healthcare use of IPV victims versus non-IPV victims, queried a large health-insured population in the northwest. The population of women studied was older and had private insurance, but researchers found women who reported IPV had 2.18 times the risk of using the ED compared to women who did not report IPV.^3^ The cohort interestingly had increased mental healthcare use, where our cohort did not demonstrate correlation of mental health complaints and increased healthcare use related to the reported offense. The conflicting results may reflect differing populations and demonstrates greater need for a population-wide prospective study characterizing types of healthcare use by IPV victims.

The third way in which our analysis is unique is the assignment of the complaint category to each visit. Assessing victims by complaint category can lead to more clinically relevant analysis when trying to identify trends in patient presentations, compared to use of discharge diagnoses. The unique data-linkage methods used here paired state law enforcement data to healthcare use and resulted in robust claims data for analysis.

While victims used healthcare services frequently up to the date of the index offense and after, there was no single complaint category that successfully identified a majority of the victims. Ascending numbers of visits up to the date of IPV-related events is supported by a three-year county-wide study.^18^ Healthcare providers may have increasing number of interactions to recognize and intervene for a victim prior to date of reported offense, but focused screening efforts cannot be supported with current research. Our paper shows that victims also came into contact with healthcare providers after IPV-related events, presenting with a myriad of complaints giving providers opportunities to identify victims. The findings in this study expand understanding of reasons victims seek medical care in the ED by demonstrating that together, obstetric-gynecologic related and infectious-related complaints represent almost half of the IPV victims’ complaints. Supporting other studies, this is evidence that non-trauma related presentations are more common than trauma- related presentations for IPV victims and suggests complaints to incorporate into IPV screening strategies.^17^,^21^ These visit patterns are key to understanding opportunities to identify IPV victims. While providers cannot focus screening strategies at this time to a specific presenting complaint, data suggest that clinicians have increasing contact with victims prior to their victimization and just after. Providers may consider adopting more in-depth screening practices for patients presenting with obstetric-gynecologic complaints. In the future, a prospective study characterizing complaints by category of IPV victims on presentation and comparing them to the non-IPV victims’ presentations could help providers recognize patterns to assist in identifying IPV victims.

## LIMITATIONS

Like most research using claims data, conclusions about the diagnostic categories and reasons for visits are limited to the researchers’ interpretation of and the strength of claims data. Retrospective data analysis also limited validity of results due to lack of control of confounding variables. The study also would have been able to make stronger conclusions about overall healthcare use had the claims data included primary care and private outpatient visits. The initial date of reported victimization in 2007 was chosen as the index offense, and it is possible that IPV occurred in prior years or after the index event in the same year. We did not analyze healthcare use trends associated with prior or repeat offenses, and this could have led to repeated measure bias. While Medicaid patients comprise an appropriate cohort for study, a larger study population across all socioeconomic categories would have strengthened external validity. Women who would not normally qualify for Medicaid can enroll when pregnant. This special population may have increased healthcare use associated with obstetric-gynecologic complaints.

## CONCLUSION

Female Medicaid-eligible IPV victims use the ED with increasing frequency as the date of the IPV abuse approaches. The women’s presenting complaints varied and did not reveal unique presenting complaints that would allow narrowing screening practices. Frequent ED use in women between the ages of 16–55 years of age should prompt healthcare providers to consider IPV.

The successful cross-referencing of administrative and legal databases suggests this is a feasible methodology in investigating other use trends surrounding other types of victimization or criminal behavior. Identifying use patterns for child abuse victims, driving under the influence offenders or suicide victims may further assist practitioners on identifying at-risk patients.
